# The feasibility and advantage of uniportal video-assisted thoracoscopic surgery (VATS) in pulmonary lobectomy

**DOI:** 10.1186/s12885-017-3069-z

**Published:** 2017-01-25

**Authors:** Linlin Wang, Dabei Liu, Jibin Lu, Suning Zhang, Xueying Yang

**Affiliations:** 1 Department of Thoracic Surgery, The Fourth Affiliated Hospital of China Medical University, No. 4, CongShan East Road, HuangGu District, Shenyang, 110032 Liaoning People’s Republic of China; 2 Department of Thoracic Surgery, Shengjing Hospital of China Medical University, No. 36 Sanhao St., HePing District, Shenyang, 110004 Liaoning People’s Republic of China

**Keywords:** Uniportal, VATS, Lobectomy, Minimally invasive surgery

## Abstract

**Background:**

Ongoing improvements in technique and instruments for video-assisted thoracoscopic surgery (VATS) have made minimally-invasive uniportal VATS lobectomy a reality. However, the outcomes of the procedure are still under investigation, and at present, uniportal VATS lobectomy is performed infrequently at most hospitals. We have therefore reviewed our outcomes with this procedure in an attempt to validate its safety, efficacy, and feasibility.

**Methods:**

We retrospectively analyzed and compared perioperative data for patients who underwent uniportal, two-port, and traditional three-port VATS lobectomy between January 2015 and December 2015 at our hospital.

**Results:**

Among 257 patients who had successful VATS lobectomy during the study period, 73 underwent uniportal VATS, 86 underwent two-port VATS, and 98 underwent traditional three-port VATS. There were no surgical or 30-day postoperative mortalities, and no significant differences in operative times, blood loss, number of lymph nodes retrieved and nodal stations explored, drainage times, length of hospital stay, or postoperative complications among the three groups. The visual analogue scale (VAS) pain scores were significantly lower in the uniportal VATS group after surgery (*P* < 0.05).

**Conclusions:**

Uniportal VATS lobectomy is a safe and feasible surgical procedure that is associated with decreased surgical trauma and less postoperative pain compared to traditional VATS. Further long term follow-up analyses in large numbers of patients are ongoing.

## Background

Rapid technological progress has allowed thoracic surgery to become more minimally invasive with faster postoperative rehabilitation [1]. Conventional three-port video-assisted thoracic surgery (VATS) lobectomy is well established, and many units have successfully adopted this technique over the past two decades [2]. Since 2010, uniportal VATS lobectomy has become a new area of exploration in minimally invasive thoracic surgery [3]. Uniportal VATS is a less invasive approach that allows major thoracic operations to be performed through a single small incision of about 4 cm. With consistent reports of excellent results, uniportal VATS is becoming more and more widely accepted [4, 5]. The advantages of uniportal VATS can include reduced surgical trauma, decreased postoperative pain, faster rehabilitation, and improved patient satisfaction with a less invasive approach than conventional VATS [6, 7]. However, concerns about operative risks, technical challenges, and unstudied outcomes of uniportal VATS lobectomy remain. Therefore, the aim of this study is to assess the feasibility and applicability of the uniportal VATS approach in pulmonary lobectomy, and to compare perioperative outcomes of uniportal VATS with those of two-port and traditional three-port VATS.

## Methods

### Data acquisition and follow-up

We retrospectively analyzed results in patients who underwent uniportal, two-port, or traditional three-port VATS lobectomy between January 2015 and December 2015 at our hospital. None of the patients had received neoadjuvant therapy before surgery. Institutional review board (IRB) approval and written informed consent were obtained. Patients underwent routine preoperative arterial blood gas analysis, pulmonary function testing, bronchoscopy, and computed tomography. Pre-, peri-, and postoperative patient details and outcome variables were collected by means of clinical assessment, patient inquiry, and review of any inpatient admissions.

### Surgical technique

Until 2014 we employed only the traditional three-port VATS technique for pulmonary lobectomy, involving 3 incisions with the operative field visualized on screen via video thoracoscope [8]. In 2014, we began performing two-port VATS lobectomy. In the two-port procedure, the video thoracoscope is introduced through the 7th or 8th intercostal space in the midaxillary line, and the operation is performed through an incision of approximately 4 cm that is placed anteriorly in the 4th or 5th intercostal space with no rib spreading. This incision serves as the utility port for the passage of instruments and staplers and ultimately for extraction of the specimen. Our substantial experience with conventional and double-port VATS prepared us to begin performing uniportal VATS lobectomies with no rib spreading beginning in January 2015.

### Uniportal video assisted thoracoscopic surgery technique

**Fig. 1:**
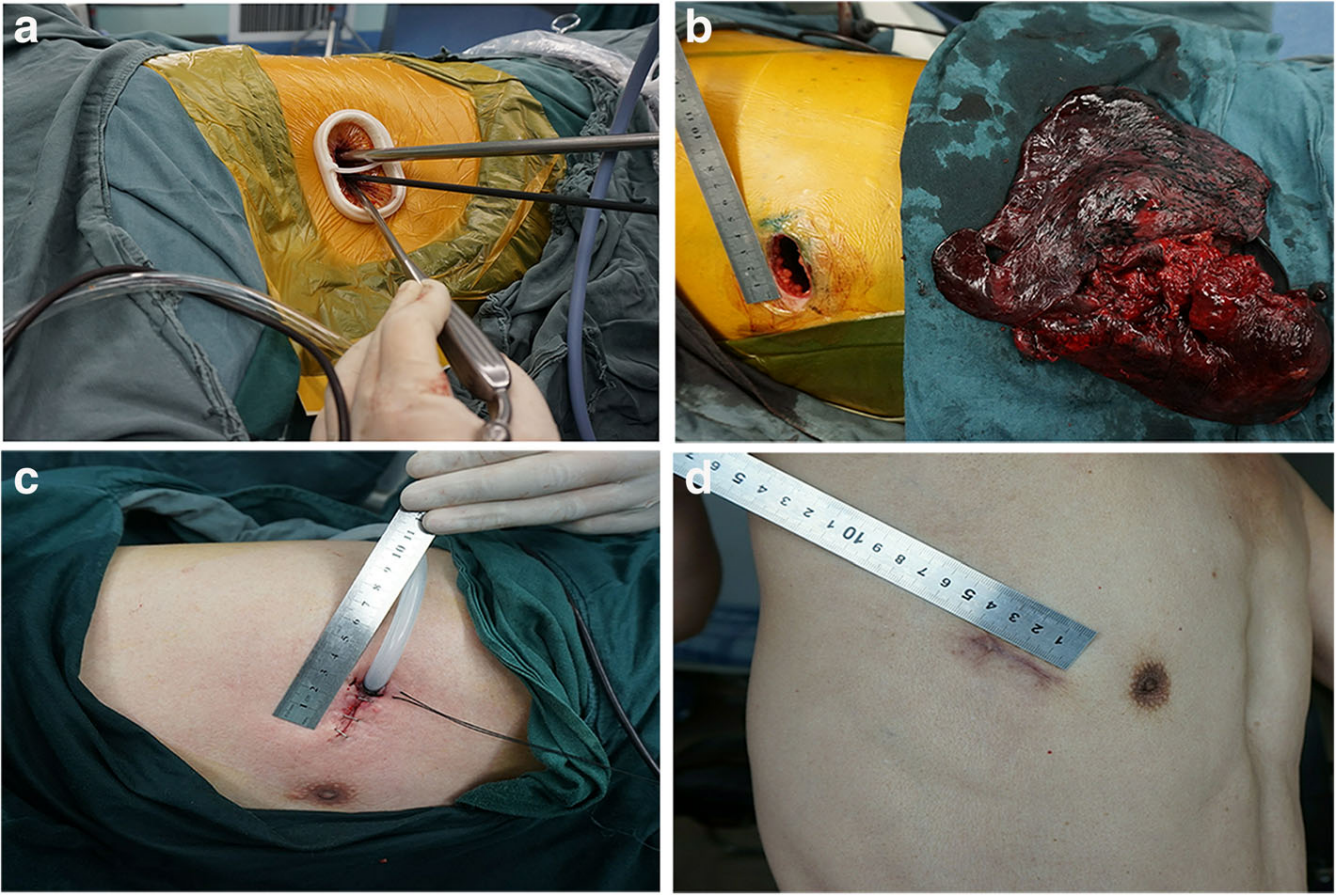
Uniportal video assisted thoracoscopic surgery (VATS) lobectomy. A single incision of approximately 4.0 cm is made in the 4th or 5th intercostal space at the anterior axillary line. **a** A specially-designed wound protector is inserted, forming two channels, one for the thoracoscope and the other for the surgical instruments. **b** Left pneumonectomy. **c** A U-shaped suture is placed around the chest tube in order to close the incision immediately after removal of the tube. **d** One month after right upper lobectomy

Uniportal VATS was performed with patients under general anesthesia with single-lung ventilation via a double lumen endotracheal tube. The patients were placed in a full lateral decubitus position with the operating table flexed to increase the intercostal spacing. A single incision of approximately 4 cm was made in the 4th or 5th intercostal space at the anterior axillary line to facilitate good access to hilar structures and lymph node stations (Fig. 1a). Both the surgeon and the assistant stood at the anterior side of the patient so that they had the same field of vision and were better coordinated. We placed a special plastic wound protector (Demai, China) in the incision to form two channels, one for the thoracoscope and the other for the surgical instruments (Fig. 1a). The thoracoscope was usually placed through the upper channel with the instruments in the lower channel, but the scope and the instruments could be easily transposed as needed. We used Ethicon (Ethicon Endo-Surgery, LLC, USA) brand instruments (i.e. Echelon Flex 45 articulating endoscopic linear cutters), as well as long curved endoscopic instruments with double articulations, curved suction (Yundi, China), and a 30° thoracoscope.

The surgeon performed the operation by bimanual instrumentation with a pair of surgical instruments that was crossed at the incision site (Fig. 1a). The surgical steps were similar to those carried out during traditional VATS, including individual dissection of the veins, arteries, and lobar bronchus and complete mediastinal lymphadenectomy, and the instrumentation for obtaining the target tissue in the single-port approach mimicked that of conventional thoracotomy [9]. We generally followed an operative sequence of vein, artery, and bronchus. However, for the left upper lobe we preferred the sequence of artery, vein, and bronchus because of the anatomical features. We used a Flex 45 endostapler (Ethicon) to manage the bronchus, incomplete fissures, and the main blood vessel, while smaller vascular branches were best addressed by silk ligature and vascular clips. Once separated, the resected lobe was placed in a protective bag and removed through the single incision (Fig. 1b). Complete mediastinal lymphadenectomy was performed in lung cancer patients, and the number of accessible lymph node stations was equivalent to that of open thoracotomy. At the end of the operation, we secured the chest tube with a U-shaped suture to allow immediate closure of the wound upon removal of the tube (Fig. 1c), and the wound was then infiltrated with ropivacaine (Naropin, AstraZeneca AB, Sweden). Lesions were localized preoperatively in patients with pulmonary ground-glass opacity (GGO) and those with small nodules by using a CT-guided puncture positioning method.

### Visual analog scale (VAS) pain scores

The intensity of postoperative pain was scored with a 10-cm vertical visual analogue scale (VAS) [10] anchored at the bottom by “no pain” and at the top by “worst imaginable pain” and marked at predetermined intervals. The patients rated their pain by marking the appropriate point between the two extremes, and the scores were determined by measuring the distance from the minimal endpoint to the mark. Pain scores were assessed at 24, 48, and 72 h postoperatively and at 1 week and 1 month after surgery.

### Statistical analysis

Categorical variables were expressed as percentages and continuous variables were expressed as mean ± standard deviation. Variables were compared using Student’s *t*-test, the *χ*2 test and variance analysis or Fisher’s exact test. Data were analyzed with SPSS 17.0 software. *P*-values < 0.05 were considered statistically significant.

## Results

### Patient characteristics

There were 257 patients who underwent successful VATS lobectomy from January 2015 to December 2015, and there were no intraoperative or 30-day mortalities. Seventy-three patients underwent uniportal VATS, 86 underwent two-port VATS, and 98 underwent traditional three-port VATS. All types and combinations of lobectomy were performed (Table 1). Postoperative pathologic diagnoses included both primary lung cancers and benign conditions (Table 2). Primary lung cancers were classified by pathologic stage (stage I, stage II, or stage III or higher; Table 3).

**Table 1:** Baseline patient characteristics

Characteristic	Uniportal VATS (*n* = 73)	Two-port VATS (*n* = 86)	Three-port VATS (*n* = 98)	*P*-value
Age (Mean ± SD)	57.12 ± 6.43	54.36 ± 7.6	61.32 ± 7.54	<0.001
Sex (%)				0.288
Male	31 (42.47%)	45 (52.33%)	53 (54.08%)	
Female	42 (51.53%)	41 (47.67%)	45 (45.92%)	
Pulmonary function (Mean ± SD)				
FVC (% predicted)	90.83 ± 13.43	88.32 ± 15.87	85.33 ± 15.87	0.064
FEV1 (% predicted)	86.18 ± 12.91	82.70 ± 16.30	80.71 ± 19.08	0.104
MVV (% predicted)	69.26 ± 18.84	67.75 ± 25.10	70.78 ± 21.43	0.649
Arterial blood gas analysis (Mean ± SD)				
PAO_2_ (mmHg)	84.87 ± 12.62	83.10 ± 18.43	79.75 ± 14.20	0.085
PACO_2_ (mmHg)	41.26 ± 5.01	42.37 ± 5.85	42.61 ± 6.12	0.286
SAO_2_ (%)	96.55 ± 1.42	95.58 ± 2.47	95.72 ± 3.37	0.048
Lobectomy (%)				
Right upper	16 (21.92%)	28 (32.56%)	23 (23.47%)	0.237
Right middle	4 (5.48%)	6 (6.98%)	11 (11.22%)	0.352
Right lower	18 (24.66%)	13 (15.12%)	23 (23.47%)	0.254
Right middle and lower	3 (4.11%)	1 (1.16%)	0 (%)	0.069
Left upper	12 (16.44%)	16 (18.6%)	24 (24.49%)	0.388
Left lower	19 (26.03%)	22 (25.58%)	16 (16.33%)	0.207
Left pneumonectomy	1 (1.37%)	0 (%)	1 (1.02%)	0.744

*FVC* forced vital capacity, *FEV*1 forced expiratory volume in 1 second, *MVV* maximal ventilatory volume, *PAO*_2_ partial pressure of oxygen in artery, *PACO*_2_ partial pressure of carbon dioxide in artery, *SAO*_2_ arterial oxygen saturation

**Table 2:** Pathology results

Pathological types	Uniportal VATS (%) (*n* = 73)	Two-port VATS (%) (*n* = 86)	Three-port VATS (%) (*n* = 98)	*P*-value
Primary lung cancer	51 (69.86%)	56 (65.12%)	71 (72.45%)	0.556
Adenocarcinoma	34 (46.58%)	38 (44.19%)	52 (53.06%)	0.459
Squamous cell carcinoma	16 (21.92%)	15 (17.44%)	13 (13.27%)	0.330
Adenosquamous	0 (0%)	1 (1.16%)	1 (1.02%)	1.000
Large cell carcinoma	1 (1.37%)	0 (0%)	2 (2.04%)	0.506
Carcinoid tumors	0 (0%)	1 (1.16%)	0 (0%)	0.619
Carcinosarcoma	0 (0%)	0 (0%)	1 (1.02%)	1.000
Small cell carcinoma	0 (0%)	1 (1.16%)	2 (2.04%)	0.780
Benign disease	22 (30.14%)	30 (34.88%)	27 (27.55%)	0.556
Inflammation	3 (4.11%)	6 (6.98%)	7 (7.14%)	0.676
Pulmonary cyst	4 (5.48%)	4 (4.65%)	4 (4.08%)	0.933
Tuberculosis	4 (5.48%)	9 (10.47%)	7 (7.14%)	0.482
Pulmonary hamartoma	2 (2.74%)	3 (3.49%)	3 (3.06%)	1.000
Pulmonary fibrosis	4 (5.48%)	3 (3.49%)	1 (1.02%)	0.223
Bronchiectasis	5 (6.85%)	4 (4.65%)	4 (4.08%)	0.723
Lung sequestration	0 (0%)	0 (0%)	1 (1.02%)	1.000
Spindle cell lipoma	0 (0%)	1 (1.16%)	0 (0%)	0.619

There were no significant difference in these results among the three groups (all *P* > 0.05)

**Table 3:** Distribution of primary lung cancers by pathologic stage

Stage	Uniportal VATS (%) (*n* = 51)	Two-port VATS (%) (*n* = 56)	Three-port VATS (%) (*n* = 71)	*P*-value
Stage I	27 (52.94%)	29 (51.79%)	34 (47.89%)	0.839
IA	13 (25.49%)	13 (23.21%)	19 (26.76%)	0.900
IB	14 (27.45%)	16 (28.57%)	15 (21.13%)	0.578
Stage II	21 (41.18%)	24 (42.86%)	30 (42.25%)	0.984
IIA	11 (21.57%)	14 (25.00%)	14 (19.72%)	0.773
IIB	10 (19.61%)	10 (17.86%)	16 (22.54%)	0.802
Stage III or greater	3 (5.88%)	3 (5.36%)	7 (9.86%)	0.625
IIIA	3 (5.88%)	3 (5.36%)	5 (7.04%)	0.928
IIIB	0 (0%)	0 (0%)	1 (1.41%)	1.000
IV^a^	0 (0%)	0 (0%)	1 (1.41%)	1.000

^a^ Isolated brain metastasis following metastasectomy prior to being evaluated for lobectomy

There were no significant difference in these results among the three groups (all *P* > 0.05)

### Surgical outcomes

There were 1, 2, and 2 procedures converted to conventional open thoracotomy in the uniportal, two-port, and three-port VATS groups, respectively, for conversion rates of 1.37%, 2.33%, and 2.04% (*P* > 0.05). Conversions were mainly due to bleeding and dense adhesions. All conversions were completed safely and no inpatient deaths occurred. There were no significant differences in operative times, blood loss, number of lymph nodes retrieved and nodal stations explored, drainage times, or length of hospital stay among the three groups (Table 4). There was no intraoperative mortality in any of the three groups. The perioperative (30-day) mortality was 0% in the uniportal and three-port VATS groups and 1.16% in the two-port VATS group, where one patient died of respiratory failure. Four patients (5.48%), 9 patients (10.47%), and 9 patients (9.18%) had postoperative complications (*P* > 0.05) (Table 5).

**Table 4:** Surgical results (Mean ± SD)

Characteristic	Uniportal VATS (*n* = 73)	Two-port VATS (*n* = 86)	Three-port VATS (*n* = 98)	*P*-value
Operative time (min)	154.88 ± 31.31	163.91 ± 49.72	162.84 ± 68.18	0.519
Blood loss (mL)	92.5 ± 22.66	100 ± 33.89	103.21 ± 27.41	0.051
Drainage time (days)	4.55 ± 1.41	5.34 ± 1.81	5.26 ± 3.15	0.069
Length of hospital stay (days)	8.63 ± 2.06	8.95 ± 2.40	9.55 ± 3.18	0.069
Number of lymph nodes retrieved (lung cancer patients only)	13.56 ± 3.79	12.68 ± 3.17	12.71 ± 4.18	0.252
Number of nodal stations explored (lung cancer patients only)	7.05 ± 1.15	6.73 ± 1.28	6.56 ± 1.49	0.059

There were no significant difference in these results among the three groups (all *P* > 0.05)

**Table 5:** Postoperative complications

Complication	Uniportal VATS (%) (*n* = 73)	Two-port VATS (%) (*n* = 86)	Three-port VATS (%) (*n* = 98)	*P*-value
Pneumonia	2 (2.74%)	3 (3.49%)	3 (3.06%)	1.000
Prolonged air leak	1 (1.37%)	2 (2.33%)	3 (3.06%)	0.879
Cardiac arrhythmias	1 (1.37%)	2 (2.33%)	1 (1.02%)	0.832
Hemothorax	0 (0%)	1 (1.16%)	2 (2.04%)	0.780
Respiratory failure	0 (0%)	1 (1.16%)	0 (0%)	0.619
Totals	4 (5.48%)	9 (10.47%)	9 (9.18%)	0.514

The overall rate of postoperative complications was no significantly differences (all *P* > 0.05) in the uniportal VATS group compared to the two- and three-port VATS groups

### Visual analogue scale pain scores

**Fig. 2:**
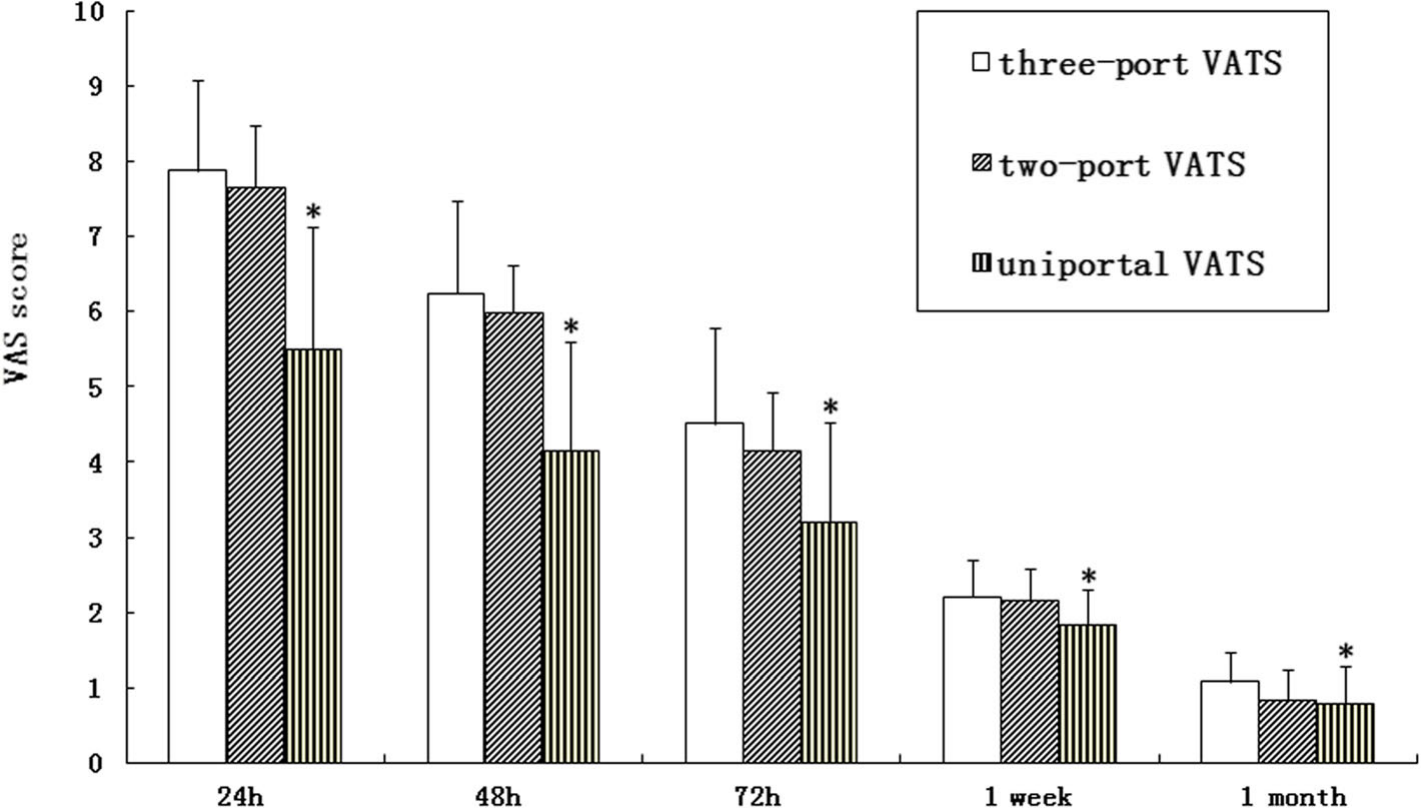
Visual analog scale (VAS) pain scores. The postoperative pain scores in the uniportal VATS group after surgery were significantly lower compared to those of the two-port and three-port VATS groups (*P* < 0.05)

In the uniportal VATS group, the mean VAS scores at 24, 48, and 72 h and 1 week and 1 month after surgery were 5.51 ± 1.62, 4.17 ± 1.44, 3.21 ± 1.32, 1.83 ± 0.47, and 0.79 ± 0.49. The VAS scores in the two-port VATS group were 7.67 ± 0.82, 6.00 ± 0.63, 4.17 ± 0.75, 2.17 ± 0.41, and 0.83 ± 0.41, and in the three-port VATS group, the scores were 7.88 ± 1.20, 6.24 ± 1.24, 4.52 ± 1.26, 2.20 ± 0.50, and 1.08 ± 0.40. The postoperative pain scores were significantly lower (*P* < 0.05) in the uniportal VATS group compared to the other two groups, confirming an advantage for uniportal VATS in terms of reduced pain during the early postoperative period (Fig. 2).

## Discussion

The surgical approach to lung resection is constantly evolving [11]. Experience gained through routine application of traditional VATS techniques combined with ongoing improvements in the surgical instruments have led to great advances in minimally invasive thoracic surgery [12]. The first uniportal VATS lobectomy was reported by Gonzalez-Rivas et al. in 2011 [3], and the technique and reliability of this approach have been improving steadily ever since [13]. Uniportal VATS has changed our outlook on minimally invasive thoracic surgery. Although it is technically demanding, once mastered, uniportal VATS can be expected to minimize the amount of surgical trauma, the advantages of which include decreased postoperative pain, shorter hospital stay, and preservation of pulmonary function, promoting more rapid recovery and providing superior cosmetic results when compared with pulmonary lobectomy via traditional three-port VATS [14, 15]. Uniportal VATS is also advantageous to the surgeon because it provides a more anatomic and direct view of the target tissues and it allows bimanual instrumentation, as in an open approach. The uniportal approach also negates the creation of torsional or dihedral angles by the instruments that is inherent to conventional multiport VATS [16].

Following the advances in video endoscopic instruments and endosurgical techniques, the indications for uniportal VATS lobectomy have been expanded to a larger patient population, and it is now a feasible option for treatment of a variety of benign lung diseases as well as for lung cancers that are amenable to complete resection by lobectomy. Greater numbers of surgeons are now gaining the experience required to perform technically demanding uniportal VATS procedures via a small single incision with excellent postoperative results [17]. Prior thoracic irradiation and induction therapy, sleeve lobectomy, and vascular reconstruction need not be considered contraindications to uniportal VATS [18], and preoperative assessment and patient selection for uniportal VATS lobectomy should be conducted as for conventional three-port VATS [19]. We are now performing almost all of our VATS lobectomies, as well as pneumonectomies, via a uniportal approach.

Uniportal VATS has been reliably comparable to multiportal VATS in terms of safety and efficacy, but the geometric configuration of the approach is completely different. Uniportal VATS is geometrically favorable to multiport techniques. It provides direct in-plane visualization of the target tissues because the thoracoscope and the surgical instruments enter the chest through the same incision. Because uniportal VATS provides the surgeon with the perspective of operating in the same projection plane and retains visual depth, it is easier to judge distances and improve the accuracy of the surgical maneuvers. However, there are also some difficulties associated with the single-incision technique. The operating instruments are more prone to impeding one another’s movements, and the assistant who is managing the thoracoscope may be more prone to fatigue and error. As mentioned, one of our techniques is to place a special plastic wound protector in the incision to form two separate channels, one for the thoracoscope and the other for the surgical instruments. This makes it possible to fix the thoracoscope in place and reduces the tendency to mutual interference of the instruments. The thoracoscope and the instruments, including the articulating endostapler and the long curved double articulation instruments, can easily be transposed for optimal positioning and angle of operation during surgery, and the demand on the assistant is lessened while the accuracy of the operation is improved. Another important issue during uniportal VATS approach is the difficulty in palpating small lung lesions through the single incision. We use a preoperative CT-guided puncture positioning method to localize small nodules and to pinpoint lesions in patients with pulmonary GGO. In addition, there has been some concern that placing the chest tube in the single incision could affect wound healing. We have addressed this by placing a U-shaped suture around the chest tube, which allows immediate closure of the wound upon removal of the tube, and have had excellent results (Fig. 1 c, d).

In this study, we compared the outcomes of pulmonary lobectomy by uniportal VATS with those of lobectomy by two- and three-port VATS and found that there were no significant differences in operative time, blood loss, number of lymph nodes retrieved and nodal stations explored, drainage time, length of hospital stay, or postoperative complications among the three groups. Rates of conversion from VATS to open surgery ranging from 2 to 23% have been reported [20]. In our study, the rates of conversion were 1.37%, 2.33%, and 2.04% for uniportal, two-port, and three-port VATS. Because discharge of our patients is delayed according to our customs, the median hospitalization period was longer compared to other hospitals, but there was no difference in length of stay among our three study groups. The average number of resected lymph nodes in our patients was lower than that in other reports [17] because we routinely perform en bloc resection of lymph nodes during mediastinal lymphadenectomy, and the number of lymph nodes retrieved and nodal stations explored did not differ among our three study groups. Finally, we found that the VAS pain scores were significantly lower after surgery in uniportal VATS group compared to the other two groups. We attributed this to reduced access trauma and reduced risk of intercostal nerve injury.

## Conclusion

Uniportal VATS lobectomy is a safe and feasible surgical procedure that is associated with reduced surgical trauma and decreased postoperative pain compared to traditional VATS. However, uniportal VATS lobectomy is a relatively complex procedure and the experience of the surgeon along with the correct instrumentation are critical to its success. Rigorous training is required for this technique, and additional long-term survival and outcomes analyses should be conducted on larger numbers of patients. We can then anticipate that uniportal VATS lobectomy will be more and more widely performed in the years to come.

## Abbreviations

CT: Computed tomography
GGO: Ground-glass opacity
IRB: Institutional review board
SD: Standard deviation
VAS: Visual analog scale
VATS: Video-assisted thoracoscopic surgery
